# The Welsh Anti-Tuberculosis Campaign

**Published:** 1915-09-18

**Authors:** 


### THE WELSH ANTI-TUBERCULOSIS CAMPAIGN.

### The Measure of the Memorial Association's Activities.

In the completeness of the machinery available for the treatment and prevention of all forms of tuberculosis the scheme now being developed in the Principality of Wales occupies a very conspicuous, if not a pre-eminent, position among anti-tuberculosis organisations. The third annual report of the King Edward VII. Welsh National Memorial Association on the work done during the twelve months ending March 31, 1915, is an unusually voluminous document. The ramifications of the Association extend throughout the thirteen counties, including Monmouth, and into the four county boroughs of Newport, Cardiff, Merthyr, and Swansea. Up to the end of March nearly 1 per cent. of the entire population in this area had been examined. The report now under notice gives full information on all details of the work of the Association, financial, administrative, and medical. The latter comprises not only numerous statistical tables, many of which, it must be admitted, are of little or no interest; but there are also included short separate reports from each of the fourteen tuberculosis physicians. These individual reports afford interesting reading, and collectively they form a supplement which may be regarded as a mirror of modern methods of dealing with tuberculous persons and the tuberculosis problem.

### The Association's Strategy.

A word must first be devoted to recalling the details of the scheme of the Association. This provides (1) for the division of the country into fourteen dispensary areas, each having a Tuberculosis Institute or headquarters and a series of visiting stations (101 in all), (2) hospitals, (3) sanatoria, and (4) educational work.

It is interesting to note that hospital treatment is intended for (*a*) cases under observation, (*b*) cases of recent origin with acute symptoms, (*c*) cases of temporary breakdown after treatment, (*d*) patients in a dying condition, (*e*) the education and training of permanently incapacitated patients. By combining all these functions it is felt that the danger of the hospital being shunned as a home for the dying should be avoided—a point to which we had recently occasion to refer in our columns.

In running through the fourteen reports of the district tuberculosis positions it becomes apparent that in all the districts a cordial relation seems to exist between the officers of the Association and the general practitioners and school doctors. As regards the patients themselves variations in their docility are only to be expected, as is evidenced by the figures relating to those who fail to continue or to come under treatment. Dr. Dan A. Powell urges the need for power of compulsory removal and detention of advanced cases in hospital. He rightly complains that, as hospital treatment increases the expectation of life of the consumptive, one result of the present system of voluntary discharge from institutions of advanced cases is to increase the infectious consumptive population of the country, and, therefore, to increase the risk to the normal population of contracting tuberculosis. It is unfortunately only too evident in numerous cases that there is no parallel between the infectiousness of the case and the precautions taken by the patient. An extended employment of skilled nurses and constant watching are necessary to diminish such a grave menace to the public health.

In this report also it is stated that the number of cases suitable for direct admission into sanatoria is still regrettably small; this indicates that the hoped-for success in getting into touch with the early case has not so far been attained.

### Points from the District Physicians' Reports.

Dr. Carveth Johnson mentions the great importance in prognosis of the mental factor, and has been struck by the very great improvement that has occurred in cases (not strictly early cases) who have made a determined fight. He also regrets the too prominent part played by tea and bread and butter in the diet in rural districts, and the too great dependence on tinned foods in industrial districts. One more interesting point calls for remark—namely, the relatively small proportion of "suspects" who eventually are proved to be suffering from tuberculosis.

Several of the reports call attention to the need for a more efficient provision of dental treatment in connection with the work of the Association. In some districts it is exceedingly difficult to get a prospective sanatoria patient's mouth put in order. On the importance of dental hygiene in the case of consumptives there are no two opinions, and an extension of the activities of the Association in this direction is looked for, especially in rural districts.

In one of the mining areas experience seems to show that men who return to underground work do better than those who prefer a "light job on top." The former have good wages and short hours; the latter usually have longer hours, less wages, and are exposed to all weathers. Some people imagine that sanatorium treatment is practically the same thing as exposure to all weathers!

The comments in this series of reports cover a very wide range; the suggestions made are not always of the most practical kind, and not a few of the *obiter dicta* are merely of the order of pious opinions. But, as we have said, this collection of reports is of great interest, as reflecting current thoughts and aims in connection with the control of tuberculosis.

### The Association's Work and the War.

Turning now to the main body of the report, we find that the efforts of the Association were not materially slackened during the year in spite of unprecedented difficulties. No fewer than ten medical officers are absent on leave serving with the forces of the Crown, and no little difficulty has been found in filling their places, but the redoubled energy of the remaining physicians, as in other depleted branches of the staff, is believed to have insured that the interests of the patients did not suffer unnecessarily. The number of hospital beds was increased during the year by 86, and there are now available 418 such beds, of which 183 are in hospitals belonging to the Association, and 235 are in institutions under the control of other authorities. Several new hospitals are in course of construction, or about to be provided, and eventually there will be a total of 686 beds belonging to the Association. On the basis suggested by the Departmental Committee on Tuberculosis, the number of beds required in Wales and Monmouth should be 478, plus 33 in respect of districts where the incidence of the disease is above normal. Experience has already shown, it is stated, that the 511 beds would be inadequate. When the scheme is fully completed, the Association will be able, if necessary, to surrender some of the beds belonging to other authorities. Of new beds 22 have already been opened for patients in Anglesey, and it is anticipated the 141 in hospitals now in course of construction will all be available during the coming autumn. In the Glan Ely Hospital, Cardiff, extensions are rapidly approaching completion, and include an operating theatre. One of the achievements of the year was the acquisition of the workhouse at Tregaron, Cardiganshire, which has been altered and equipped as a tuberculosis hospital with 32 beds, and as the central dispensary for the county. The cost was £2,497.

Beechwood House, the loan of which by the Newport County Borough Council has been reported upon, will be ready in a few weeks for the reception of some fifty sailors, soldiers, or munition workers. It will be recalled that there was considerable discussion at the time as to whether the accommodation should be restricted to sailors and soldiers only (THE HOSPITAL, July 31, 1915, p. 365).

### THE POSITION IN PEMBROKE.

At the annual meeting of the Board of Governors, held at Llandudno last month, the presidential address by Lieut.-Colonel David Davies, M.P., referred to the disappointment which the position in Pembroke had caused the Association. The County Council has refused to reimburse the Association for all the work done in that area, and it apparently has some idea of starting a scheme of its own, but nothing substantial seems as yet to have come of the proposal. Since July 1912 the Association has provided treatment to all persons suffering from tuberculosis in the county, a service which has cost £2,000 over and above the contributions of the Pembrokeshire Insurance Committee. It was stated that the County Council had actually levied a rate and collected the money to defray this expense, but not a penny had been handed over. The Association apparently not only loses in this way the sum equal to half the deficiency which the County Council is asked to pay, but the other half, payable by the Treasury, will not be contributed to the funds of the Association. Henceforth the uninsured consumptives of Pembroke will not be able to obtain treatment except by paying for it out of their own pockets. From the correspondence it appears that the County Council has displayed no little obstinacy in its attitude towards the Association, and in its last communication it decided no longer to appoint representatives to the Board of Governors or the Council of the Association. The Welsh Memorial Association has continued gratuitous treatment for nearly three years and is now left with a bad debt. With all the other County Councils the most cordial relations have prevailed.

### THE £150 PER BED "ILLUSION."

The evidence of the report points to the fact that the Welsh National Memorial Association during the last twelve months has made good progress with its ambitious schemes, especially in regard to the provision of hospitals and sanatoria. Such constructive work has been seriously hampered, and, in the coming year, may be even more difficult to carry through. A remark in the president's address will not fail to interest hospital workers: "It has been proved to the satisfaction of everybody that it is impossible to provide institutions at a cost not exceeding £150 per bed." The Treasury grant in aid of building hospitals will not exceed £56 per bed, in the case of sanatoria the sum will be £80. A higher figure was hoped for, but the incidence of the disease is such that the amount available for Wales will not allow of a bigger distribution.

### DR. MARCUS PATERSON'S VIEWS.

The report of the Medical Director, Dr. Marcus Paterson, in spite of several points of interest, can only briefly be alluded to here. He emphasises the view that tuberculosis is as much a social as a medical problem, and particularly deplores the fact that the consumptive who is careful enough to use a sputum flask in public is deliberately shunned, while his fellow-sufferer who uses the dangerous handkerchief or even spits on the floor is sympathetically tolerated. This state of affairs is a grave reflection on the lay Press of the country, for it is certainly through the incomplete education of the public by newspaper articles that the widespread phobia of tuberculosis has reached such an absurd pitch as it undoubtedly has done, while the lessons taught by the careful consumptive have not been generally learnt. Only the other day a doctor in the medical Press was bemoaning the way in which his own daughter, a sufferer from phthisis, was treated by those who ought to know better.

The work which the Association has set itself is being carried on with laudable thoroughness, but it may be anticipated that the medical part at any rate will have to be seriously curtailed during the next twelve months on account of the urgent need for medical men in the new Armies. It is perhaps not unjustifiable to suggest that the anti-tuberculosis campaign, either in Wales or elsewhere, will not suffer as much as many enthusiasts would have us believe as the result of such a curtailment.

